# Seroprevalence of hepatitis B and C viruses and risk factors in HIV infected children at the felgehiwot referral hospital, Ethiopia

**DOI:** 10.1186/1756-0500-7-838

**Published:** 2014-11-25

**Authors:** Bayeh Abera, Yohanes Zenebe, Wondemagegn Mulu, Mulugeta Kibret, Getachew Kahsu

**Affiliations:** Department of Medical Microbiology, Parasitology and Immunology, College of Medicine and Health Sciences, Bahir Dar University, Bahir Dar, Ethiopia; Department of Biology, Science College, Bahir Dar University, Bahir Dar, Ethiopia; Bahir Dar Regional Health Research Laboratory Center, Bahir Dar, Ethiopia

**Keywords:** HBV, HCV, HIV, ALT, Children

## Abstract

**Background:**

Liver hepatitis due to Hepatitis B (HBV) and hepatitis C virus (HCV) co-infection is the leading cause of morbidity and mortality in HIV infected children and it is more severe in resource poor settings. Data on seroprevalence of HBV and HCV among HIV infected children are scarce in Ethiopia. This study was conducted to determine seroprevalence and risk factors of HBV and HCV and its effect on liver enzyme among HIV-positive children aged 18 months to 15 years attending the paediatric HIV care and treatment clinic at Felege Hiwot referral hospital, Ethiopia.

**Methods:**

A cross-sectional study was conducted in May, 2014. Demographic and risk factors were collected using a structured questionnaire. Hepatitis B surface antigen (HBsAg) and anti-HCV antibodies were detected using an enzyme linked immunosorbent assay (ELISA). Alanine aminotransferase (ALT) levels were determined. The results were analyzed using descriptive and logistic regression.

**Results:**

A total of 253 HIV positive children, boys (52.5%) and girls (47.5%) took part in the study. The median age of the children was 11 years. Overall, 19 (7.5%) of HIV infected children were positive either for HBsAg or anti-HCV antibodies. The seroprevalence of HBV and HCV were 2.0% and 5.5%, respectively. All HBsAg positive children were in older age groups (11-15years). Seroprevalence of HCV was higher in children from urban (7.7%) than rural (1.2%) residents (P = 0.02). Overall, 29 (12.1%) of children had elevated ALT. Of these, 31.5% were from HBsAg or anti-HCV antibody positive children whereas 9.8% were from hepatitis B or C virus negative children (P = 0.001). Multivariate logistic regression showed that being positive for HBsAg or anti-HCV antibody (AOR: 4.7(95% CI: 1.5-13.5) was significantly associated with elevated ALT.

**Conclusion:**

HBV and HCV co-infections are common in HIV positive children. In HIV positive children, HBV and HCV co-infection were associated with elevate ALT. Routine screening for HBV and HCV in HIV infected children should be implemented.

## Background

Co-infection of HIV positive population with hepatitis B (HBV) and hepatitis C (HCV) is a global problem owing to the shared routes of transmission. Prevention and control of these infections are more challenging in sub-Saharan Africa [1]. In Ethiopia, 1.3 million people live with HIV (78% adults and 22% children < 14 years) [2]. Although, the exact prevalence of HIV in children in Ethiopia is not known, approximately, 154,084 children live with HIV in 2014 and 16,000 children were receiving ART in 2013 [2].

Globally, over 350 and 170 million people are chronically infected with HBV and HCV, respectively [3, 4]. In sub-Saharan Africa, the overall prevalence of HBV and HCV co- infection in HIV infected people were 15% and 7%, respectively [5]. In Ethiopia, virtually, all data on sero-prevalence of HBV and HCV have been documented from adults [6, 7].

Both HBV and HCV infections cause acute and chronic liver infection with potential to liver cirrhosis and hepatocellular carcinoma [8]. Studies showed that HBV and HCV infections are the major causes of morbidity and mortality in HIV positive population related to liver cirrhosis and hepatocelular carcinoma [9, 10]. The risk for development of liver cirrhosis is twice in HCV/HIV co-infected than HIV mono infected groups [11]. A study conducted in Thailand showed that HBV and HCV co-infection accounted for increased mortality in ART-naïve patients [12].

WHO recommends screening for HBV and HCV before initiating ART [13]. However, in Ethiopia screening for HBV and HCV in HIV positive individuals is not practiced. For instance, a recent study in the same area reported 6.6% HIV prevalence and 19.0% HBV-HIVco-infection in pregnant women, respectively [14]. There is a paucity of seroprevalence data on HBV and HCV in HIV infected children in Ethiopia. This study was therefore undertaken to determine seroprevalence and risk factors of HBVand HCV and its effect on liver ALT among HIV infected children aged 18 months to 15 years at Felege Hiwot referral hospital, Ethiopia.

## Methods

### Study design and setting

A hospital based cross-sectional study was conducted among HIV infected children at Felege hiwot referral hospital in May 2014. The hospital is one of the government-sponsored ART centers in Bahir Dar city. This hospital provides chronic HIV care (both pre-ART and ART) services. At the time of study, there were 1, 810 HIV positive children (810 Pre-ART and 1000 on ART) HIV at Felege hiwot pediatric ART clinic [personal communication]. The choice of ART was based on the Ethiopian ART guidelines. The three first line regimens generally comprised of [Tenofovir (TDF) + Lamivudine (3TC) + Nevirapine (NVP)/Efavirenz (EFV); or Zidovudine (AZT) +3TC + NVP/EFV; or Stavudine (d4T) +3TC + NVP/EFV] [15].

### Sample size and sampling technique

Sample size was determined using Epi info version 7.0 (public domain soft ware http://www.cdc.gov) considering 95% confidence level, marginal error of (5%) and 10% prevalence of HBsAg [1]. Thus, sample size of 239 was calculated and10% (n = 24) was added for non-respondents. Random sampling technique was used to enroll the study participants using their registration logbook.

### Sample collection and processing

A pre-tested questionnaire was used to collect data on demographic characteristics and associated risk factors such as sex, age, duration of ART, history blood transfusion, female genital mutilation (FGM), uvulectomy and residence of by face to face interviewing the childrens’ parent. The presence of hepatitis B surface antigens (HBsAg) and anti-HCV antibody (IgG and IgM) were detected on sera using ELISA according to the manufacturer’s instruction (Linear Chemicals. Joaquim Costa, Barcelona, Spain). The catalytic activity of ALT (alanine amino transferase) was determined in serum using Beckman Coulter Synchron Clinical Systems auto lab analyzer (Beckman Coulter Inc. Fullerton, USA). Normal value for ALT at 37°C (0–40 IU/L) was taken for both females and males. Laboratory quality controls were maintained by following standardized procedures during blood sample collection, storage and analytical process. Internal positive and negative controls were run alongside of the test.

### Statistical analysis

Data was analyzed using the Statistical Package for Social Sciences (SPSS® 20, USA). All explanatory variables with a p value ≤0.2 in the bivariate analysis were included in the multivariate logistic regression model to identify independent predictive variables. Odds ratio (OR) and 95% confidence intervals (CI) were calculated and the result was considered statistically significant at p < 0.05. Fisher’s exact test was used to test categorical variables.

### Ethical consideration

The study was ethically approved by the Research and Ethical Review Board of Bahir Dar University. Moreover, all parents of children gave written consent to participate in this study. Children positive for HBSAg and ant-HCV antibody were reported to ART clinic pediatrician for further investigation and follow-up.

### Operational definition of liver toxicity degree according to WHO

*Degree* 0: the level of toxicity considered normal when its value is >1.25 × normal value of ALT.

*Degree* 1: the level of toxicity considered as weak when its value is 1.256 – 2.5 × normal value of ALT.

*Degree* 2: the level of toxicity considered as moderate when its value is 2.6 – 5 × normal value of ALT.

*Degree* 3: the level of toxicity considered as severe when its value is 5.1 – 10 × normal value of ALT.

*Degree* 4: the level of toxicity considered as severe when its value is >10 × normal value of ALT.

## Results

### Demographic characteristics

A total of 253 HIV infected children participated in the present study. Among these, 133 (52.5%) were boys and 120 (47.5%) were girls. The median age of the children was 11 years. Of these, 87.7% of children were on ART. The median length of time on ART was 48 months. During the study period, 96.7% of children were on the first line ART regimen. Demographic characteristics of hepatitis co-infection in children are summarized in Table 1.

**Table 1:** **Seroprevalence of HBV and HCV and demographic characteristics among HIV infected children**, **North West Ethiopia**, **2014**

Characteristics	Total sample	HBSAg positive N (%)	HCV antibody positive N (%)	Total N (%)
**Sex**				
Female	120	2 (1.7)	4 (3.3)	6 (5.0)
Male	133	3 (2.2)	10 (7.5)	13 (9.7)
**Total**	**253**	**5** (**2.0**)	**14** (**5.5**)	**19** (**7.5**)
**Age group** **(years)**				
≤5	14	0 (0)	1 (7.1)	1 (7.1)
6-10	103	0 (0)	4 (3.9)	4 (3.9)
11-15	136	5 (3.6)	9 (6.6)	14 (10.2)
**Total**	**253**	**5** (**2.0**)	**14** (**5.5**)	**19** (**7.5**)

### Seroprevalence of hepatitis B and C viruses

Overall, 19 (7.5%) HIV infected children were positive either for HBsAg or ant-HCV antibodies. The seroprevalence of HCV and HBV were 5.5% and 1.2%, respectively. No study participant was positive for both HBV and HCV. All HBsAg positive children were in older age groups between 11–15 years of age (Table 1). Hepatitis co-infected children were significantly older than their HIV-mono infected counter parts (P = 0.01) (Table 2). The seroprevalence of HCV was higher in children from urban than rural residents (P = 0.02). However, statistical significance difference was not observed between HBV prevalence and age groups. The results of the investigated potential risk factors are depicted in Table 2. Overall, significant association was not observed for seroprevalence of hepatitis and any of the investigated risk factors (Table 2).

**Table 2:** **Association of local risk factors for HBV and HCV co**-**infection in HIV infected children**, **North West**, **Ethiopia**, **2014**

Variables	Total sample	HBSAg positive N (%)	P value	HCV seropositive N (%)	P value
**Sex**					
Girls	120	2 (1.7)	0.56	4 (3.3)	0.14
Boys	133	3 (2.2)		10 (7.5)	
**Mean age** **(years)**	11.1 ± 3.4**	13.4 ± 2.7	0.001	12.5 ± 4.1	0.01
**Residence**					
Urban	169	2 (1.2)	0.16	13 (7.7)	0.02
Rural	84	3 (3.5)		1 (1.2)	
**Uvulectomy**					
Yes	53	0 (0)	NA	5 (9.4)	0.16
No	200	5 (2.5)		9 (4.3)	
**Blood transfusion**					
Yes	9	0 (0)	NA	1 (1.1)	NA
No	244	5 (2.0)		13 (5.3)	
**FGM**					
Yes	25	0 (0)	NA	1 (4.0)	NA
No	228	5 (2.2)		13 (5.7)	
**WHO staging**					
1 and 2	173	4	0.41	9	0.46
3 and 4	80	1		5	

**: Mean age for mono HIV infection; NA: not applicable, FGM: female genital mutilation.

### Associated risk factors

According to WHO classification of HIV stage, 31.6% of children were either in stage 3 or 4 HIV (Table 2). In this study, 25 (20.8%) of the girls had female genital mutilation (FGM) and 53 (21%) had traditional uvulectomy. Nine participants (3.5%) had history of blood transfusion. Among those with history of uvulectomy, 9.4% of them were positive for anti-HCV antibody. Among blood transfusion recipients, 1/9 (1.1%) was positive for anti-HCV antibody. Conversely, none of them were positive for HBsAg.

### Alanine amino transferase (ALT)

Among 222 children on ART, 29 (12.1%) had elevated ALT compared to 2 (6.4%) among 31 on pre-ART groups (Table 3). The proportion of elevated ALT among HBsAg or anti-HCV antibody positive children was 31.5%. But, 9.8% elevated ALT was from those without hepatitis co-infection. Nineteen (7.5%) of participants had grade 1 and 2 liver toxicity. Of these, only three children (1.2%) developed grade 2 liver toxicity. However, none of the study participants developed either grade 3 or 4 liver toxicity. Multivariate logistic regression analysis showed that being positive for HBsAg or anti-HCV antibody (AOR: 4.7(95%CI: 1.5-13.5) was significantly associated with elevated ALT (Table 3).

**Table 3:** **Bivariate and multivariate logistic regression for associated variables for elevated ALT**

Variables	Elevated ALT N(%)	Univariate COR 95% CI	Multivariate AOR (95% CI)
**Sex**			
Female (n = 120)	10 (9.0)	0.5 (0.22-1.30)	0.36 (0.03-2.7)
male (n = 133)	19 (14.3)		
**ART status**			
On ART (n = 222)	27 (12.2)	1.6 (0.35-7.3)	2.0 (0.43-6.8)
Not on ART (n = 31)	2 (6.4)	1.0	
**Residence**			
Urban (n = 169)	16 (9.4)	1.0	
Rural (n = 84)	13 (15.4)	0.51 (0.22-1.2)	3.2 (1.2-8.2)*
**HBSAg**			
Positive (n = 5)	3 (60)	1.9 (0.22-18.1)	0.49 (0.06-4.3)
Negative (n = 248)	26 (10.5)	1.0	
**Anti**-**HCV Ab**			
Positive (n = 14)	3 (21.4)	2.3 (0.4-10.0)	1.6 (0.22-11.5)
Negative (n = 239)	26 (5.8)	1.0	
**Anti**-**HCV or HBsAg**			
Positive (n = 19)	6	3.2 (1.4-14.2)	4.7 (1.5-13.5)**
Negative (n = 234)	23	1.0	

*: P value 0.01 and **P value = 0.001; ART: antiretroviral therapy; AOR: adjusted odds ratio; COR: crud odds ratio; anti-hepatitis C virus antibodies; CI: confidence interval.

## Discussion

The present study demonstrated the prevailing seroprevalence of HBV and HCV in HIV infected children for the first time in Ethiopia. The antiretroviral therapy (ART) program in Ethiopia has significantly reduced morbidity and mortality in HIV infected individuals [16]. However, liver diseases from HBV and HCV co-infection may have negative impact on the success of ART program particularly in resource poor settings where facilities are limited for HBV and HCV testing and monitoring.

The HBsAg prevalence in HIV infected children in this study (2.0%) is comparable with studies in Thailand (3.3%), Democratic Republic of Congo (1.6%) and Tanzania (1.2%) and [12, 17, 18]. In contrast, it is lower compared to other studies in Africa. For instance, 5.8% to 15.9% of HBV/HIV sereoprevalence in HIV infected children was reported in West Africa [19–22]. Moreover, it is far lower than the HBsAg prevalence (11.2% to 19.0%) among HIV positive adults in the same study area in Bahir Dar [14, 23]. To our knowledge, there are no data on HBsAg in HIV positive children in Ethiopia therefore we could not compare our results directly with similar age groups.

This low HBsAg prevalence in children cannot be attributed to HBV vaccine program since all study participants had not been vaccinated. The other possible factors may be occult hepatitis B virus infection (OBI) because OBI is common in HIV infected children [24]. Furthermore, the effect of the potential anti-hepatitis B virus agent lamivudine (3TC) may be attributed to low prevalence in HBV. Because, 87.7% of HIV infected participants were on ART that contain 3TC [15]. The investigated local risk factors such as female genital mutilation (FGM), uvuloctomy, blood transfusion and WHO HIV staging had no significant association with prevalence of HBsAg. The only associated risk factor for HBsAg positive was age of the participants (P = 0.001) (Table 2). Likewise, studies in a Nigeria and USA reported that HBV co-infection was associated in children aged 11–15 years [20, 25].

The seroprevalence of HCV in this study (5.5%) conforms to the estimated prevalence of HCV in Africa (5.3%), which is the highest in the world [26]. It is also comparable with reports from Nigeria (6.8%) [27]. Comparison was not possible in Ethiopian context owing to lack of HCV seroprevalence data in HIV infected children. Although, the study population is different, previous studies in Ethiopia reported 4.5 to 9.2% HCV seroprevalence in HIV positive adults [28, 29]. In contrast, in other studies 13.5% and 10.3% seroprevalence of HCV were reported in HIV-infected children [16, 30].

In this study, local risk factors for hepatitis co-infection were investigated hence 20.8% of girls had female genital mutilation (FGM). This is similar with the national data which stated that 25% of 0–14 years old children had FGM [31]. But, in our study none of them were found to be positive for either of the hepatitis virus markers. Although, statistical significant association was not noted, children who have had history of traditional uvulecyomy had higher HCV seroprevalnce (9.4%) than their counterparts without history of uvulecyomy (4.3%). In this study, seroprevalence of HCV was higher in children from urban (7.7%) than rural children (1.2%) (P = 0.02). The other investigated potential risk factors were not significantly associated with seroprevalence of HCV or HBV (Table 2).

Regards to ALT, liver toxicity was mild since none of the participants developed either grade 3 or 4 liver toxicity. However, 7.5% of children developed grade 1 and 2 of liver toxicity. ART usage had no significant association with elevated ALT. Similarly, in Tanzanian children no association was documented between usage of ART and elevated ALT [18]. This may be elucidated by duration of ART and in this study 96.7% of children were on the first line ART regimen during the study period. Using multivariate logistic regression analysis, children positive either for HBsAg or anti-HCV antibody had 4.7 times more risk of elevated ALT (Table 3).

This study was not without limitation thus detecting of active replication of HCV and its genotype in anti-HCV patients may be more accurate than antibody test but were not possible due to the limitations of working in resource poor settings [32]. However, these data showing a high rate of HCV co-infection highlight the urgent need for more detailed characterization HCV by HCV RNA testing and HCV genotyping. Thus, preliminary information on HCV has paramount importance to plan resources to avail the emerging curative treatment for HCV using direct acting antiviral agents (DAAs) that are associated with high (>95%) cure rates.

## Conclusion

Overall seroprevalence of hepatitis B and C viruses in HIV positive children were high. Hepatitis co-infections in HIV positive children were associated with elevated ALT. Therefore, routine screenings for HBV and HCV in HIV positive children need to be in place.

## Authors’ information

BA is associate professor of Medical Microbiology, in the department of Medical microbiology, Immunology and Parasitology, at College of Medicine and Health Sciences, Bahir Dar University, Ethiopia. YZ is lecturer in the department of Medical microbiology, Immunology and Parasitology, at College of Medicine and Health Sciences, Bahir Dar University, Ethiopia. WM, is assistant professor in the department of Medical microbiology, Immunology and Parasitology, at College of Medicine and Health Sciences, Bahir Dar University, Ethiopia. MK, is associate professor of applied Microbiology Department of Biology, Science College, Bahir Dar University, Ethiopia. GK, is Bench scientist at Bahir Dar Regional Health Research Laboratory Center, Amhara region, Ethiopia.
